# Contemplative Practices: A Strategy to Improve Health and Reduce Disparities

**DOI:** 10.3390/ijerph15102253

**Published:** 2018-10-15

**Authors:** Marino A. Bruce, Kia Skrine Jeffers, Jan King Robinson, Keith C. Norris

**Affiliations:** 1 Program for Research on Faith and Health, Center for Research on Men’s Health, Vanderbilt University, Nashville, TN 37240, USA; 2 Center for Medicine, Health, and Society, Vanderbilt University, Nashville, TN 37240, USA; 3 Department of Population Health Science, John D. Bower School of Population Health, University of Mississippi Medical Center, Jackson, MS 39201, USA; KCNorris@mednet.ucla.edu; 4 National Clinician Scholars Program at UCLA, Los Angeles, CA 90095, USA; kiajeffers@ucla.edu; 5 School of Nursing at UCLA, Los Angeles, CA 90095, USA; 6 Former Healthcare Chief Operating Officer and Interim CEO, Advisory Board, N.C. Eastern Area Health Education Center, Vice Chair, Board of Trustees, Elizabeth City State University, Elizabeth City, NC 27909, USA; jkrob810@gmail.com; 7 David Geffen School of Medicine at UCLA, Los Angeles, CA 90095, USA

**Keywords:** spirituality, meditation, health disparities, stress reduction

## Abstract

Health has many dimensions, and intolerance and lack of compassion may contribute to the poor health and disparities in our nation. Tolerance can convey an inherent paradox or dissonance that can be associated with stress. However, tolerance has a dimension of acceptance, an acknowledgement and acceptance of what “is” at the present moment, that can relieve tension associated with differing beliefs and practices. Compassionate consideration of others can be combined with acceptance to create harmony within and across individuals. In this article, we explore how contemplative practices can cultivate tolerance and compassion and contribute to improvements in individual and population health.

## 1. Introduction

In 2017, Dr. Robert Brook wrote an editorial in JAMA where he posed a provocative question, “Should the Definition of Health Include a Measure of Tolerance?” [1]. In other words, should a community or nation be considered healthy if hatred is pervasive, and likewise should an individual be considered healthy if he or she is hateful and intolerant? “The use of the word tolerance has undergone many changes in its meaning. Miriam-Webster has two definitions for tolerance: (1) the capacity to endure pain or hardship, and (2) sympathy or indulgence for beliefs or practices differing from or conflicting with one’s own. Tolerance therefore “contains an inherent paradox of accepting the things one rejects or objects to” [2] (p. 1). On the surface, this may not appear to be a good thing. Inherent paradox, the dissonance between one’s actual condition vs. one’s desired condition closely resembles stress, and prolonged stress leads to deleterious health outcomes. The inherent paradox of tolerance is distinctively different from stress in that tolerance involves acceptance. It is an acceptance of people and an acceptance of what “is” in the present moment. Acceptance quiets the tension of the inherent paradox. Indeed, at the dedication of the Museum of Tolerance, Federico Mayor, Director General of the United Nations Educational, Scientific and Cultural Organization (UNESCO) emphasized that valuing one another “is the ethical basis for peace, security and intercultural dialogue.” By contrast, compassion is a sense of our feeling as another and desiring to alleviate their suffering. Together they can be a powerful force for creating harmony and social/health equity. In this paper, we explore how contemplative practices might cultivate tolerance and compassion leading to active listening and more authentic patient-provider interactions. Trained clinicians can collect religious or spiritual history information and encourage patients to connect with organizations that cultivate and/or promote tolerance and compassion. These evidence-based practices can transform us as individuals such that we have a greater sensitivity toward and reverence of our health and those around us to ultimately influence individual and population level health. Health disparities are pronounced between different race/ethnicity categories and socioeconomic levels. Contemplative practices address disparities because they can be utilized in culturally meaningful ways (i.e., the patient can use approaches such as prayer or meditation that are most meaningful to them), and they can be implemented at no or low cost. Contemplative practices can also empower people who are disadvantaged, marginalized, or underresourced to respond to the stresses that surround them (e.g., racism, poverty) in ways that serve their spiritual, emotional, mental, and physical health needs.

## 2. Health

The World Health Organization (WHO) defined health as a “state of complete physical, mental and social well-being and not merely the absence of disease or infirmity” [3]. An expanded understanding of health can be gained by considering its etymology. The word “health” derives from the old English word root “hal or halig”, which actually means to be whole or holy (https://www.etymonline.com/word/health) [4]. Taken together with the WHO definition, health encompasses synchrony within and between individuals—i.e., health encompasses a connection between: (a) physical and mental conditions that are associated with one’s senses (seeing, hearing, smelling, touching, tasting), (b) personal experiences, which may be physical/mental or expressed less tangibly through one’s soul/spirit, and (c) one’s social condition, which is shaped through one’s interactions with others. Thus, health implies a state of interconnectedness. Health represents the integration of physical, mental, and spiritual well-being within an individual who is also part of a collective, social whole. In our view, tolerance and compassion emerge from the individual and collective awareness of our interconnectedness, and the degree to which we treat others as beings to whom we are interconnected influences our health as well as theirs.

## 3. Contemplative Practices

Examples of holistic approaches to health can be found in societies in which contemplative practices are normalized. Deep contemplation of self, environment, and spirit can raise awareness of humans being connected to a greater whole. Common forms include meditation (e.g., transcendental meditation, contemplative meditation, breathing meditation), mindfulness, Tai Chi/Qigong, yoga and prayer, often practiced twenty minutes or more, once or twice daily, but can extend into daily life as well. Some patients may find some practices to be more or less culturally appropriate. Any contemplative practice that resonates for them should be encouraged. It is estimated that 8% of adults in the United States practice some form of meditation, and as many as 24% of patients with cardiovascular disease (CVD), for example, have used some form of mind-body therapy [5]. An increasing number of contemplative practices are being used as “health” interventions for individuals with select health conditions. Recent systematic reviews of meditation and mindfulness-based interventions have shown contemplative practices to be effective for multiple conditions including, but not limited to, cardiovascular disease, post-traumatic stress, vascular disease, fibromyalgia, and lower back pain [5,6,7,8,9]. Thus, contemplative practices may influence health at the individual as well as the population level.

## 4. Spiritual Awareness

“A human being is a part of a whole, called by us ‘universe’ a part limited in time and space. He experiences himself, his thoughts and feelings as something separated from the rest... a kind of optical delusion of his consciousness. This delusion is a kind of prison for us, restricting us to our personal desires and to affection for a few persons nearest to us. Our task must be to free ourselves from this prison by widening our circle of compassion to embrace all living creatures and the whole of nature in its beauty.” (Albert Einstein) [10]

Spiritual awareness refers to a state of being, and it is accessible across contemplative practices. Spiritual customs, habits, or rituals including contemplative practices are often vehicles to heightened awareness and insight. When individuals are in a state of increased awareness, they are conscious of their thoughts and emotions and move from the reactive mind to the responsive mind. They are able to perceive experiences with more clarity than when their thoughts are clouded by conditioning and/or intolerance. Awareness frees individuals to experience people and circumstances as they are without judgment or the confines of preconceived ideas, which Albert Einstein noted in the quote above are “a kind of optical delusion” [10] which is a “prison for us”. One of the goals of contemplative practices is to extend the periods of time during which we are in a state of increased awareness, recognizing our connectedness with ourselves and others, collectively present in space and time. Individual and collective awareness fosters tolerance through the mechanism of compassion.

## 5. Compassion

**Figure 1:**
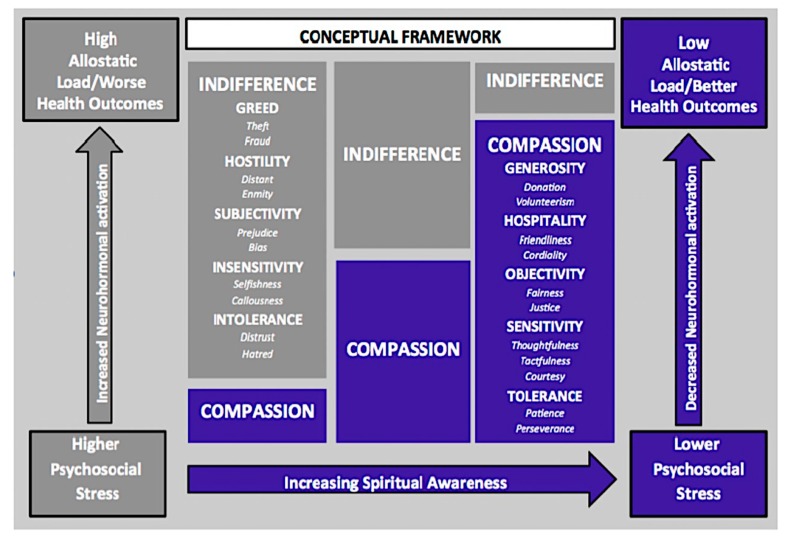
Legend. A proposed framework by which increasing spiritual awareness may reduce psychosocial stress and allostatic load to improve health outcomes.

Compassion is often a product of enhanced spiritual awareness. Awareness and compassion work together to decrease both intolerance and stress. Research shows that compassion may mediate some of the psychosocial health benefits of contemplative practices [11,12], including reducing stress [13]. Figure 1 depicts a schema that shows a proposed process by which spiritual awareness may lead to lower psychosocial stress (belief or thought that demands and expectations being placed on one exceed their ability to cope [14,15,16,17,18,19], decreased neurohormonal activation, low allostatic load (the level of wear and tear on the body that accumulates as an individual is exposed to repeated or chronic stress [19]), and better health outcomes. The process is mediated by decreased intolerance and increased compassion. Contemplative practices may play an important role in this process. A discussion about contemplative practices and stress can be found in the next section.

## 6. Contemplative Practices and Stress in the Context of Health and Disease

Stress involves environmental, social, or internal demands that result in physiological and/or emotional arousal that can have physical and/or psychological consequences [14,15,16,17,18]. Stress can activate neurohormonal systems that control the body’s response to threatening situations [19] (e.g., a flight or fight reaction in response to a physical threat). This critical system allows for survival in response to external threats. However, when this state of activation is disproportionate to the situation it becomes maladaptive and counterproductive, and can produce undesired health outcomes. Examples include increased blood pressure, premature heart disease, increased inflammation, and immunologic dysfunction [20,21,22,23,24,25,26,27,28,29].

By creating a state of greater harmony and balance within one’s body, contemplative practices can mitigate maladaptive biological (e.g., elevated blood pressure) and behavioral (e.g., substance abuse) responses, and select medical conditions (e.g., depression). Contemplative practices can also foster an opportunity to raise one’s awareness about stressful, underlying issues [30,31]. Contemplative practices have been particularly useful in the treatment of post-traumatic stress disorder [9], in cardiovascular risk reduction [5,32,33], reducing health care provider burnout by increasing self-compassion [34], and possibly prolonging longevity [35,36].

**Figure 2:**
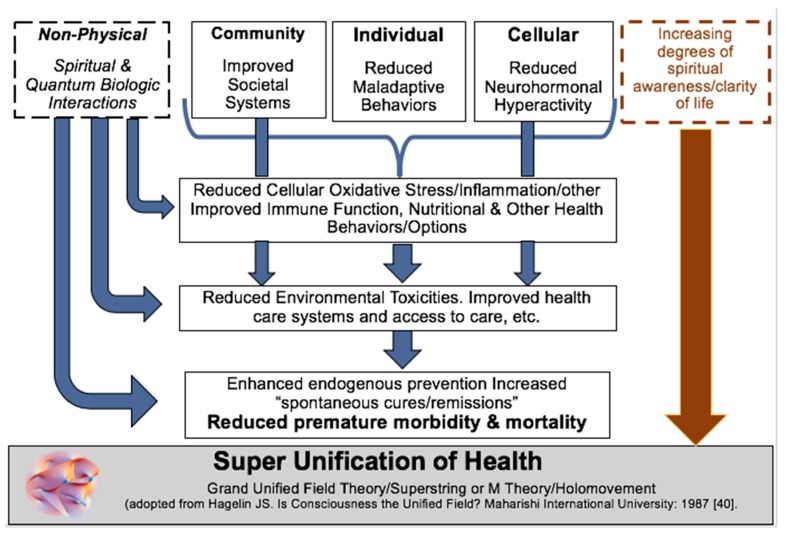
Legend: Four proposed major domains of health: (**1**) spiritual and quantum interactions; (**2**) community; (**3**) individual; and (**4**) cellular all interact through multiple pathways to reach a space where all four operate in synchrony, analogous to super-unification of the major forces of nature within physics [41]. Superimposed with this is the vision that contemplative practices may enhance spiritual awareness or a sense of oneness to advance us along the path toward a space where there may emerge a state of super unification of health.

Mechanistically, contemplative practices have been noted to mediate health outcomes at a cellular level through its effects on gene expression [37,38,39]. A conceptual model of how contemplative practices may improve health through the effects of progressive levels of consciousness is shown in Figure 2. The increase of awareness (particularly spiritual awareness or sense of oneness) on four fundamental domains of health is viewed analogous to the Grand Unification Theory [40] or super unification [41] in physics where there is a space in which the four major forces of nature (gravity, electromagnetism, strong and weak force) can be considered to act as one force. Similarly, we posit that with increasing levels of spiritual awareness, as cultivated through contemplative practices, a state may be achieved where each of the four fundamental domains of health operate as one based on awareness of the whole. Hagelin described this state as transcendental consciousness [41]. While the concept of unification of energy is often studied in physics [40,41], it is rare to conceptualize different domains of biology and health as part of a larger whole. Yet, in daily life, such concepts are embraced and possibly enacted through activities such as intercessory prayer [42], while in biology it may be one of the mechanisms involved in self-organization [43]. This can be summed up in terms of a notion introduced by Bohm called quantum wholeness, which implies that the world cannot be precisely analyzed into independently and separately existent parts [44]. Contemplative practices enhance our awareness of oneness and can move us closer to that space where all aspects of health from community to cellular to quantum biologic merge into one space that might be considered super unification of health (Figure 2).

## 7. The Way Forward: In What Way Might Contemplative Practices Improve Health and Reduce Health Disparities?

Most studies suggest the possible benefit of contemplative practices on health. Given the low costs and low risks, this approach may be considered by those interested in focusing more on health prevention and/or as a non-pharmacologic approach to treating many disease states alongside other interventions such as lifestyle modification or western allopathic pharmacologic interventions. If we include tolerance/compassion as part of our assessment of health, contemplative practices will have an even more central role in our health care system and promoting a culture of health. Contemplative practices appear to have a positive impact on not only individual health, but by extending the level of compassion and tolerance beyond self, can improve health at a public/societal level and reduce disparities.

Health disparities are unlikely to be eliminated without such a sense of tolerance/compassion and caring to move us to not only aspire to, but to demand a greater level of equity in society. As health care providers continue to lead the way in delivering high-quality services to patients, an increase in their awareness can extend their impact by taking religious or spiritual histories using tools such as the CSI-MEMO, ACP, or FICA spiritual history instruments [45] during visits and partnering with religious and spiritual-oriented organizations to ensure that all patients, regardless of class or nationality, have opportunities to experience multiple dimensions of care and caring that contribute to a healthy life. Truly achieving improved health for all will require a tolerant and compassionate society, and with increased awareness and clarity comes the action to build the requisite systems to promote social and health equity, as well as the economic transformation needed to improve the lives of future generations [46]. A progressive increase in prescribing contemplative practices as a low- or no-cost, self-empowering tool for increasing spiritual awareness and improving health and self-reliance could help to reach low income, disenfranchised and often racial/ethnic minority communities that suffer most from societal-based stresses and high rates of treatable health disparities [47,48,49,50].

Increasing spiritual awareness from contemplative practices can extend the focus of U.S. health care to include with equipoise the needs of all citizens, from those who are wealthy and insured to those with less who are often un- or underinsured and underserved. Such a state of tolerance/compassion and selflessness that is cultivated by contemplative practices is captured by this quote attributed to Pema Chrodron, “Compassion is not a relationship between the healer and the wounded. It's a relationship between equals. Only when we know our own darkness well can we be present with the darkness of others. Compassion becomes real when we recognize our shared humanity” [51].

## References

[1] Brook R.H. (2017). Should the definition of health include a measure of tolerance?. JAMA.

[2] Van Doorn M. (2014). The nature of tolerance and the social circumstances in which it emerges. Curr. Soc..

[3] World Health Organization Constitution of WHO: Principles. https://www.who.int/about/mission/en/.

[4] Online Etymology Dictionary Health. https://www.etymonline.com/word/health.

[5] Levine G.N., Lange R.A., Bairey-Merz C.N., Davidson R.J., Jamerson K., Mehta P.K., Michos E.D., Norris K., Ray I.B., Saban K.L. (2017). Meditation and cardiovascular risk reduction: A scientific statement from the american heart association. J. Am. Heart Assoc..

[6] Abbott R.A., Whear R., Rodgers L.R., Bethel A., Thompson Coon J., Kuyken W., Stein K., Dickens C. (2014). Effectiveness of mindfulness-based stress reduction and mindfulness based cognitive therapy in vascular disease: A systematic review and meta-analysis of randomised controlled trials. J. Psychosom. Res..

[7] Anheyer D., Haller H., Barth J., Lauche R., Dobos G., Cramer H. (2017). Mindfulness-based stress reduction for treating low back pain: A systematic review and meta-analysis. Ann. Intern. Med..

[8] Lauche R., Cramer H., Dobos G., Langhorst J., Schmidt S. (2013). A systematic review and meta-analysis of mindfulness-based stress reduction for the fibromyalgia syndrome. J. Psychosom. Res..

[9] Cushing R.E., Braun K.L. (2018). Mind-body therapy for military veterans with post-traumatic stress disorder: A systematic review. J. Altern. Complement. Med..

[10] Albert Einstein: “A Human Being is Part of A Whole, Called by us the ‘Universe’ —A Part Limited in Time and Space”. https://www.wildmind.org/blogs/quote-of-the-month/quote-einstein-connectedness.

[11] Lawler-Row K.A., Elliott J. (2009). The role of religious activity and spirituality in the health and well-being of older adults. J. Health Psychol..

[12] Koenig H.G., George L.K., Titus P. (2004). Religion, spirituality, and health in medically ill hospitalized older patients. J. Am. Geriatr. Soc..

[13] Sprecher S., Fehr B. (2006). Enhancement of mood and self-esteem as a result of giving and receiving compassionate love. Curr. Res. Soc. Psychol..

[14] Bruce M.A., Griffith D.M., Thorpe R.J. (2015). Stress and the kidney. Adv. Chron. Kidney Dis..

[15] Cohen S., Kessler R.C., Gordon L.U., Cohen S., Kessler R.C., Gordon L.U. (1995). Personality characteristics as moderators of the relationship between stress and disorder. Measuring Stress: A Guide for Health and Social Scientists.

[16] Holmes T.H., Rahe R.H. (1967). The social readjustment rating scale. J. Psychosom. Res..

[17] Thoits P.A. (1995). Stress, coping, and social support processes: Where are we? What next?. J. Health Soc. Behav..

[18] Thoits P.A. (2010). Stress and health: Major findings and policy implications. J. Health Soc. Behav..

[19] McEwen B.S. (1998). Protective and damaging effects of stress mediators. N. Engl. J. Med..

[20] Auchincloss A.H., Diez Roux A.V., Brown D.G., O’Meara E.S., Raghunathan T.E. (2007). Association of insulin resistance with distance to wealthy areas: The multi-ethnic study of atherosclerosis. Am. J. Epidemiol..

[21] Black P.H. (2003). The inflammatory response is an integral part of the stress response: Implications for atherosclerosis, insulin resistance, type II diabetes and metabolic syndrome X. Brain Behav. Immun..

[22] Clark R., Benkert R.A., Flack J.M. (2006). Large arterial elasticity varies as a function of gender and racism-related vigilance in black youth. J. Adolesc. Health.

[23] Harris C.W., Edwards J.L., Baruch A., Riley W.A., Pusser B.E., Rejeski W.J., Herrington D.M. (2000). Effects of mental stress on brachial artery flow-mediated vasodilation in healthy normal individuals. Am. Heart J..

[24] Kop W.J., Verdino R.J., Gottdiener J.S., O’Leary S.T., Bairey Merz C.N., Krantz D.S. (2001). Changes in heart rate and heart rate variability before ambulatory ischemic events. J. Am. Coll. Cardiol..

[25] Kovach J.A., Nearing B.D., Verrier R.L. (2001). Angerlike behavioral state potentiates myocardial ischemia-induced T-wave alternans in canines. J. Am. Coll. Cardiol..

[26] Lind L., Johansson K., Hall J. (2002). The effects of mental stress and the cold pressure test on flow-mediated vasodilation. Blood Press..

[27] Rosmond R. (2005). Role of stress in the pathogenesis of the metabolic syndrome. Psychoneuroendocrinology.

[28] Williams J.E., Nieto F.J., Sanford C.P., Couper D.J., Tyroler H.A. (2002). The association between trait anger and incident stroke risk: The atherosclerosis risk in communities (ARIC) study. Stoke.

[29] Williams J.E., Nieto F.J., Sanford C.P., Tyroler H.A. (2001). Effects of an angry temperament on coronary heart disease risk: The atherosclerosis risk in communities study. Am. J. Epidemiol..

[30] Blom K., Baker B., How M., Dai M., Irvine J., Abbey S., Abramson B.L., Myers M.G., Kiss A., Perkins N.J. (2013). Hypertension analysis of stress reduction using mindfulness meditation and yoga: Results from the harmony randomized controlled trial. Am. J. Hypertens..

[31] Kabat-Zinn J., Lipworth L., Burney R. (1985). The clinical use of mindfulness meditation for the self-regulation of chronic pain. J. Behav. Med..

[32] Schneider R.H., Grim C.E., Rainforth M.V., Kotchen T., Nidich S.I., Gaylord-King C., Salerno J.W., Kotchen J.M., Alexander C.N. (2012). Stress reduction in the secondary prevention of cardiovascular disease: Randomized, controlled trial of transcendental meditation and health education in Blacks. Circ. Cardiovasc. Qual. Outcomes.

[33] Schneider R.H., Castillo-Richmond A., Alexander C.N., Myers H., Kaushik V., Aranguri C., Norris K., Haney C., Rainforth M., Calderon R., Nidich S. (2001). Behavioral treatment of hypertensive heart disease in African Americans: Rationale and design of a randomized controlled trial. Behav. Med..

[34] Gergen Barnett K.A. (2017). In pursuit of the fourth aim in health care: The joy of practice. Med. Clin. N. Am..

[35] Diamond S.J., Thomas C.R., Desai S., Holliday E.B., Jagsi R., Schmitt C., Enestvedt B.K. (2016). Gender differences in publication productivity, academic rank, and career duration among U.S. academic gastroenterology faculty. Acad. Med. J. Assoc. Am. Med. Coll..

[36] Bruce M.A., Martins D., Duru K., Beech B.M., Sims M., Harawa N., Vargas R., Kermah D., Nicholas S.B., Brown A. (2017). Church attendance, allostatic load and mortality in middle aged adults. PLoS ONE.

[37] Buric I., Farias M., Jong J., Mee C., Brazil I.A. (2017). What is the molecular signature of mind-body interventions? A systematic review of gene expression changes induced by meditation and related practices. Front. Immunol..

[38] Duraimani S., Schneider R.H., Randall O.S., Nidich S.I., Xu S., Ketete M., Rainforth M.A., Gaylord-King C., Salerno J.W., Fagan J. (2015). Effects of lifestyle modification on telomerase gene expression in hypertensive patients: A pilot trial of stress reduction and health education programs in african americans. PLoS ONE.

[39] Epel E.S., Puterman E., Lin J., Blackburn E.H., Lum P.Y., Beckmann N.D., Zhu J., Lee E., Gilbert A., Rissman R.A., Tanzi R.E., Schadt E.E. (2016). Meditation and vacation effects have an impact on disease-associated molecular phenotypes. Transl. Psychiatr..

[40] Ellis J.R., Tamvakis K., Nanopoulos D.V. (1982). Grand unification in simple supergravity. Phys. Lett. B.

[41] Hagelin J.S. (1987). Is consciousness the unified field? A field theorist’s perspective. Conscious. Based Educ. Phys..

[42] Aviles J.M., Whelan S.E., Hernke D.A., Williams B.A., Kenny K.E., O’Fallon W.M., Kopecky S.L. (2001). Intercessory prayer and cardiovascular disease progression in a coronary care unit population: A randomized controlled trial. Mayo Clin. Proc..

[43] Coffey D.S. (1998). Self-organization, complexity and chaos: The new biology for medicine. Nat. Med..

[44] Bohm D. (2002). Wholeness and the Implicate Order.

[45] Koenig H.G. (2013). Spirituality in Patient Care: Why How, When, and What.

[46] Kim J.Y., Chan M. (2013). Poverty, health, and societies of the future. JAMA.

[47] Escoto K.H., Milbury K., Nguyen N., Cho D., Roberson C., Wetter D., McNeill L.H. (2018). Use of complementary health practices in a church-based African American cohort. J. Altern. Complement. Med..

[48] Spears C.A., Houchins S.C., Bamatter W.P., Barrueco S., Hoover D.S., Perskaudas R. (2017). Perceptions of mindfulness in a low-income, primarily African American treatment-seeking sample. Mindfulness.

[49] Zernicke K.A., Campbell T.S., Speca M., McCabe-Ruff K., Flowers S., Dirkse D.A., Carlson L.E. (2013). The eCALM Trial-eTherapy for cancer applying mindfulness: Online mindfulness-based cancer recovery program for underserved individuals living with cancer in Alberta: Protocol development for a randomized wait-list controlled clinical trial. BMC Complement. Altern. Med..

[50] Schneider R.H., Walton K.G., Salerno J.W., Nidich S.I. (2006). Cardiovascular disease prevention and health promotion with the transcendental meditation program and Maharishi consciousness-based health care. Ethn. Dis..

[51] Chödrön P. (2007). The Places that Scare You: A Guide to Fearlessness in Difficult Times.

